# Bioinformatics as a Tool for Assessing the Quality of Sub-Cellular Proteomic Strategies and Inferring Functions of Proteins: Plant Cell Wall Proteomics as a Test Case

**DOI:** 10.4137/bbi.s2065

**Published:** 2009-02-18

**Authors:** Hélène San Clemente, Rafael Pont-Lezica, Elisabeth Jamet

**Affiliations:** Surfaces cellulaires et Signalisation chez les Végétaux, UMR 5546 CNRS-UPS-Université de Toulouse, Pôle de Biotechnologie Végétale, 24 chemin de Borde-Rouge, BP 42617 Auzeville, 31326 Castanet-Tolosan, France.

**Keywords:** bioinformatics, cell wall, plant, proteomics

## Abstract

Bioinformatics is used at three different steps of proteomic studies of sub-cellular compartments. First one is protein identification from mass spectrometry data. Second one is prediction of sub-cellular localization, and third one is the search of functional domains to predict the function of identified proteins in order to answer biological questions. The aim of the work was to get a new tool for improving the quality of proteomics of sub-cellular compartments. Starting from the analysis of problems found in databases, we designed a new *Arabidopsis* database named *ProtAnnDB (http://www.polebio.scsv.ups-tlse.fr/ProtAnnDB/)*. It collects in one page predictions of sub-cellular localization and of functional domains made by available software. Using this database allows not only improvement of interpretation of proteomic data (top-down analysis), but also of procedures to isolate sub-cellular compartments (bottom-up quality control).

## Introduction

**Figure 1.**
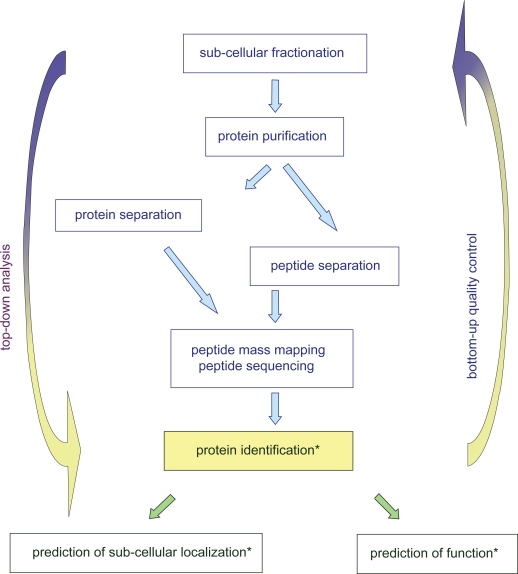
**Bioinformatic tools in proteomic strategies**. Different steps are required for protein identification in complex samples from sub-cellular fractionation to mass spectrometry analysis. Bioinformatics can be used for three different purposes indicated by stars: protein identification, prediction of protein sub-cellular localization, and prediction of protein function.

Bioinformatics is of paramount importance for protein analysis in proteomic studies. Proteomics generates huge amounts of data that must be interpreted to answer biological questions. It is used at three steps of proteomic studies (Fig. 1): (i) identification of proteins by peptide mass mapping or peptide sequencing; (ii) prediction of their sub-cellular localization, (iii) prediction of their function. Identification of proteins requires measurement of mass/charge ratios of tryptic peptides or of ions resulting from peptide fragmentation and comparison to sequence databases. This part of the work is done with devoted software such as ProteinProspector (http://prospector.ucsf.edu/), Mascot (http://www.matrixscience.com/search_form_select.html), and Profound (http://prowl.rockefeller.edu/prowl-cgi/profound.exe) that can be used online. Of course, reliability of such identifications depends on the quality of both sequences deposited in databases and of structural annotation of genomes. But many efforts have been done to improve it, especially with the setting of Unigene at NCBI which is defined as “An Organized View of the Transcriptome” (http://www.ncbi.nlm.nih.gov/sites/entrez?db=unigene). Analysis of protein sub-cellular localization and function is more puzzling. For *Arabidopsis* proteins, information on sub-cellular localization can only be found at MIPS (http://mips.gsf.de/proj/plant/jsf/athal/searchjsp/index.jsp). Information on prediction of functional domains is given in most databases. However, scores are not always given and the names of proteins may not be related to these predictions. The availability of all this information in a reliable and friendly way appeared critical when we obtained loads of data from proteomics. We wanted to use bioinformatics not only as a tool to interpret our experimental data in a “top-down analysis”, but also as “bottom-quality control” of our procedure for preparation of plant cell walls (Fig. 1)^1^,^2^. Starting from the analysis of problems found in databases, we designed a new *Arabidopsis* database named *ProtAnnDB* for *Protein Annotation DataBase*. It collects predictions made by available software. It allows the user to see the results in one page without any need to run their query against all of them.

## Prediction of Sub-Cellular Localization of Proteins as a Valuable Tool to Assess the Quality of Sub-Cellular Proteomics

The creation and maintenance of cellular structures relies on the regulated expression and spatial targeting of proteins. In addition, determining the sub-cellular localization of a protein is an important first step towards understanding its function. Proteomics is one of the large-scale methods used to identify proteins, made possible by the sequencing of whole genomes. The homogenized cells are fractionated, most commonly through centrifugation. With proper purification and fractionation techniques, the contents of a particular fraction will correspond to a particular organelle. However, the approach is sensitive to contaminations. Purity control mainly relies on tests that should be positive for proteins expected in the purified fraction, and negative for proteins of all other cell compartments.

Plant cell wall is a particular difficult compartment since it is not surrounded by a membrane, and contains anionic carbohydrates that can bind basic proteins from other compartments. The reliability of protein profiling for a compartment like the cell wall thus strongly depends on the quality of the extraction protocol. There are two ways to obtain proteins from the cell wall compartment:^4^–^5^ (i) non-destructive methods using the culture medium of cell cultures, or the extraction of extra-cellular fluids by an infiltration/centrifugation procedure; (ii) destructive methods involving the rupture of the cells and the separation of the insoluble cell wall fraction. Since plant cell walls are mainly built up with highly dense polysaccharides, this property can be used to purify them by centrifugation through high density solutions. Several methods have been used to prepare enriched cell wall fractions and to verify their purity (Table 1). On the one hand, enzymology, immunology, and microscopy have been used to estimate the purity of the isolated fraction. On the other hand, bioinformatic analyses of the sequences of identified proteins allowed the prediction of their sub-cellular localization using PSORT (http://psort.ims.u-tokyo.ac.jp/form.html)^6^ and TargetP (http://www.cbs.dtu.dk/services/TargetP/).^7^,^8^ In a few cases, sub-cellular localization could not be reliably predicted. The ratio of the number of predicted secreted proteins to the total number of proteins identified has been calculated. This ratio can also be used as a purity control. It can be seen that classical methods of control support a high purity for most of the fractions. However, the concrete results of the bioinformatic predictions show a different picture with much lower degree of purity in most cases, suggesting that many fractions were not pure enough. On the contrary, although such controls have not been performed in the case of preparation of proteins from the hypocotyl cell wall fraction,^1^ it is remarkable that the level of purity of the cell wall protein fraction is one of the highest as calculated from bioinformatic predictions. It should be noted that the use of biochemical or immunological markers as purity criteria have produced a number of publications claiming that many well-known intracellular proteins are also secreted without a signal peptide.^9^ It is true that not all secreted proteins contain a signal peptide and alternative secretion pathways exist in both prokaryotes and eukaryotes. However, only a reduced number of proteins are secreted without a signal peptide in eukaryotes.^10^

These comparisons show that the classical methods used to test for the purity of sub-cellular compartments are not conclusive for proteomic studies. Indeed, the sensitivity of mass spectrometry is much higher than that of enzymatic or immunological tests using specific markers. As a consequence, the characterization and prediction of the intrinsic signals that target proteins to the correct subcellular compartment has become a major task in bioinformatics. Although not all signals for protein sorting in cell compartments are described, bioinformatics can help in predicting subcellular localization of proteins thus contributing to the quality control of proteomic strategies (Fig. 1). In particular, sorting signals for vacuoles are of several types and probably not all are known.^11^ In addition, non classical pathway for protein secretion should be taken into account.^10^

## Using Functional Domains as Efficient Tools for Annotation of Proteins

**Figure 2.**
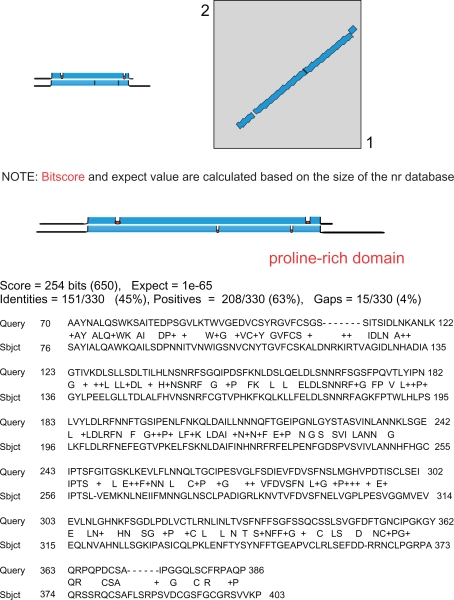
**BLAST 2 sequences alignment between amino acid sequences of *At2g19780* and *At3g24480***. BLAST was done using BLAST 2 sequences (http://blast.ncbi.nlm.nih.gov/bl2seq/wblast2.cgi). Query stands for amino acid sequence of *At2g19780* (402 amino acids). Subject stands for amino acid sequence of *At3g24480* (494 amino acids). Note that there is 45% identity and 63% similarity between the LRR regions. The proline-rich domain of *At3g24480* is outside of this alignment at the C-terminus of *At3g24480.*

With regard to protein function and due to automatic annotation of proteins on the basis of BLAST searches (http://blast.ncbi.nlm.nih.gov/Blast.cgi),^12^ there are many mistakes in databases on the principle of the children game called the Chinese whispers. Even if functional domains such as InterPro, PFAM or PROSITE are now indicated in the description of protein sequences in most databases, the names proposed for proteins are often incorrect because they result from BLAST searches rather than from the presence of functional domains. Actually, BLAST results can rely on partial sequence homology as shown in the case of the family of 11 leucine-rich repeat extensins (LRXs)^13^ as LRXs and PEXs. Query of the NCBI Entrez Protein database (http://www.ncbi.nlm.nih.gov/sites/entrez?db=Protein) results in 14 accession numbers using the following key words: leucine-rich repeat AND extensin AND Arabidopsis. The same functional annotation was found at TAIR (http://arabidopsis.org/index.jsp) and TIGR (http://www.tigr.org/tdb/e2k1/ath1/) whereas only 6 proteins were given related names such as leucine-rich repeat/extensin or extensin-like at MIPS (http://mips.gsf.de/proj/plant/jsf/index.jsp) (Table 2). A detailed analysis of the information available in databases shows that the appropriate functional domains are listed in the description of the proteins (Table 1, supplementary data). However, the names assigned to the proteins are not correct at NCBI, TAIR, and TIGR in three cases (At2g19780, At4g06744, and At4g29240) since these names were given according to BLAST results. As shown for At2g19780 in Figure 2, significant identity was found with an LRX protein encoded by *At3g24480*, but only in its leucine-rich repeat (LRR) domain. All proteins that are *bona fide* LRXs according to Baumberger et al.^13^ should have at least one LRR domain and one proline-rich domain (Table 3). Annotation of At2g19780, At4g06744, and At4g29240 should be revised. On the contrary, At2g19780 and At3g24480 are annotated as “disease resistance proteins” at MIPS since many of such proteins have LRR domains. But there is no experimental evidence that these two proteins play any role in plant defense. At present, an annotation mentioning only the presence of structural LRR domains would be more relevant.

Other problems result from different annotations despite the presence of identical functional domains. This is the case for the extensin gene family which comprises 19 members.^14^ Three examples are given in Table 2, supplementary data. At1g21310 gets the name of its mutant (RSH for Root Shoot Hypocotyl Defective) whereas At1g26240 is annotated as “proline-rich extensin-like family protein” and At1g26250 as “proline-rich extensin, putative.” On the contrary, the same name can be attributed to proteins that belong to different families. The “Pollen Ole e1 allergen and extensin family” contains two types of proteins (Table 2, supplementary data): proteins that only contain the IPR006041 domain (Pollen Ole e1 allergen and extensin), and proteins that contain both this domain and a proline-rich region profile (PS50099). Only the MIPS database names At3g33790 a “putative proline-rich protein.”

Although functional domains are correctly listed in description of proteins, annotation may only take into account a profile with a high probability of occurrence, i.e. of poor significance. This is the case of At2g15770 annotated as “glycine-rich protein” although the score obtained for PS50315 (glycine-rich region) is very low (Table 2, supplementary data). A higher score was obtained for IPR008972 (cupredoxin) and the gene has been annotated as AtEn23 by experts.^15^ On the contrary, At1g15825 has been annotated as “hydroxyproline-rich glycoprotein family protein” although the only identified structural domain is PS50099 (proline-rich region). A “BLAST 2 sequences” reveals a very poor matching to the true extensin mentioned in databases (At3g19020) (data not shown). Moreover, the prediction for subcellular localization is “cytoplasm” using PSORT with a score of 0.450 and “other” using TargetP with a score of 0.602. A detailed analysis of the protein sequence reveals that at present the only possible annotation is “proline-rich protein”.

**Figure 3.**
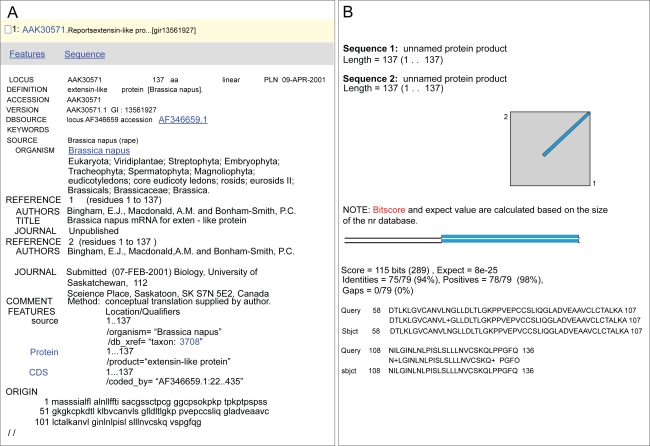
**Functional annotation of *At1g12090* in databases deduced from that of AAK30571. A**) Description of the AAK30571 sequence at the NCBI protein database (http://www.ncbi.nlm.nih.gov/sites/entrez?db=Protein). **B**) BLAST was done using BLAST 2 sequences (http://blast.ncbi.nlm.nih.gov/bl2seq/wblast2.cgi). Query stands for amino acid sequence of *At1g12090* (137 amino acids). Subject stands for amino acid sequence of AAK30571 (137 amino acids).

The last case will concern a protein that has been annotated as an “extensin-like protein” as inferred from sequence comparison to a protein sequence deduced from a cDNA of *Brassica napus* (AAK30571) (Fig. 3A). Again, the relevant functional domain is indicated in databases, i.e. IPR003612 (plant lipid transfer protein/seed storage/trypsin-alpha amylase inhibitor). The “BLAST 2 sequences” against AAK30571 gives 94% identities (Fig. 3B). However, since the annotation of the *B. napus* sequence is wrong, this mistake has been spread over other sequences that are also wrongly annotated.

## Building of the *ProtAnnDB* Dedicated to Collection of Predictions of Sub-Cellular Localization and Functional Domains

The *Arabidopsis* protein sequences present in *ProtAnnDB* (http://www.polebio.scsv.ups-tlse.fr/ProtAnnDB/) are taken from the latest version of the *Arabidopsis* genome annotation (TAIR8, http://www.arabidopsis.org/help/helppages/BLAST_help.jsp#datasets) (32825 proteins).^16^ *ProtAnnDB* provides a link to the NCBI reference protein sequences (RefSeq) which are curated sequences (http://www.ncbi.nlm.nih.gov/RefSeq/).^17^ Amino acid sequences have been run against different software predicting either their sub-cellular localization or functional domains. Different programs were used because each of them has its own specificity and it is necessary to compare the results to increase confidence in the prediction. There are feature-based methods, global sequence properties-based methods, and machine learning methods such as neural networks, hidden Markov models and support vector machines.^8^

**Figure 4.**
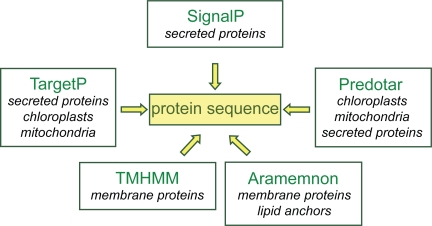
Bioinformatics tools for prediction of protein sub-cellular localization. Protein sequences are from TAIR8 (http://www.arabidopsis.org/index.jsp). **Aramemnon**: http://aramemnon.botanik.uni-koeln.de/ **Predotar**: http://urgi.versailles.inra.fr/predotar/predotar.html **SignalP**: http://www.cbs.dtu.dk/services/SignalP/ **TargetP**: http://www.cbs.dtu.dk/services/TargetP/ **TMHMM**: http://www.cbs.dtu.dk/services/TMHMM-2.0/

Sub-cellular localization of proteins can be predicted using several programs on line. TargetP, SignalP (http://www.cbs.dtu.dk/services/SignalP/),^10^ and Predotar (http://urgi.versailles.inra.fr/predotar/predotar.html)^18^ predict N-terminal targeting sequences. TMHMM (http://www.cbs.dtu.dk/services/TMHMM-2.0/) predicts transmembrane domains. Since all these programs use different algorithms to make predictions, it is necessary to compare the results. There is no such tool available on line apart from Aramemnon (http://aramemnon.botanik.uni-koeln.de/), a database describing plant putative membrane proteins based on the comparison of results from different programs predicting transmembrane spanning domains and membrane-anchoring through GPI anchors, prenylation, and myristoylation.^19^ Several of these programs were selected in *ProtAnnDB*: SignalP, TargetP, Predotar, TMHMM and Aramemnon (Fig. 4).

**Figure 5.**
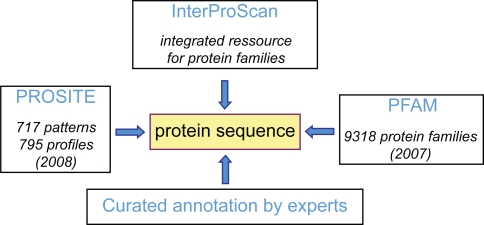
Bioinformatics tools for prediction of cell wall protein functional domains. Protein sequences are from TAIR8 (http://www.arabidopsis.org/index.jsp). Links to NCBI RefSeq are provided for each protein (http://www.ncbi.nlm.nih.gov/RefSeq/). Examples of cell wall-related gene families annotated by experts are listed in Table 4. **InterProScan**: http://www.ebi.ac.uk/Tools/InterProScan/ **PFAM**: http://pfam.sanger.ac.uk/search?tab=searchSequenceBlock **PROSITE**: http://www.expasy.org/prosite/

Prediction of functional domains can be achieved using different programs. InterProScan (http://www.ebi.ac.uk/Tools/InterProScan/) collects information from different prediction programs and proposes classification of proteins in superfamilies.^20^ Pfam (http://pfam.sanger.ac.uk/) comprises 9318 protein families.^21^ PROSITE (http://www.expasy.org/prosite/) comprises 717 patterns and 795 profiles.^22^ PANTHER (http://www.pantherdb.org/) classifies genes by their functions, using published experimental evidence and evolutionary relationships. Three of these programs were chosen for building *ProtAnnDB*: InterProScan, PROSITE and PFAM (Fig. 5).

In all cases, use of several programs based on different rationales is required to improve the quality of the prediction of sub-cellular location or function of a protein. However, some doubtful cases cannot be resolved. Finally, relevant literature, especially if it includes experimental data, is the best source of information. Databases annotated by experts in the field also provide checked information and often allow use of unified nomenclature. This information is available either in research articles or on line. It has been included in *ProtAnnDB* for 1565 sequences of cell wall-related proteins, such as (i) proteins involved in cell wall biogenesis or metabolism, and (ii) proteins predicted to be at the cell wall/plasma membrane interface like receptor-like kinases (Table 4).

**Figure 6.**
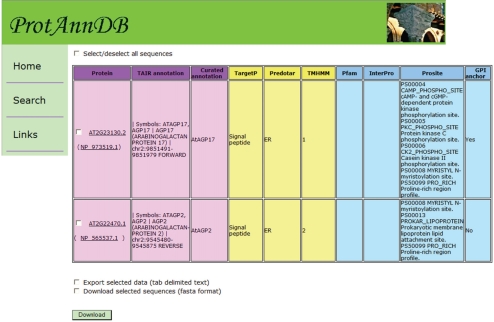
**Output of** ***ProtAnnDB*** **after query with two cell wall arabinogalactan protein AGI codes (*At2g23130*,** ***At2g22470*).** The table comprises several columns: on the left side, the three mauve columns contain the AGI code of the gene and the RefSeq accession number of the protein, the TAIR annotation, and the curated annotation, respectively; in the central part, the four yellow columns contain results of prediction of sub-cellular localization by TargetP, Predotar, and TMHMM, as well as prediction of presence of GPI anchor taken from Aramemnon; on the right part, the three blue columns contain results of prediction of functional domains by PFAM, InterProScan and PROSITE. By a click on each column head, it is possible to get an explanation on the result. It is also possible to download the protein sequences in the FASTA format and the content of the whole table in a Microsoft Office Excel compatible format.

**Figure 7.**
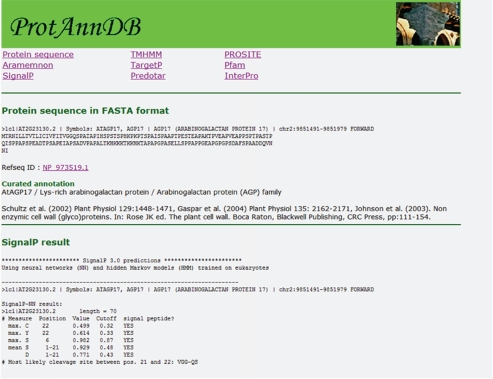
**Output of** ***ProtAnnDB*** **showing detailed results of predictions of sub-cellular localization and of presence of functional domains for the cell wall arabinogalactan protein encoded by** ***At2g23130***. Only the upper part of the web page is shown. A menu offers the possibility to quickly reach the results of prediction with the different software. The first heading collects the protein sequence in FASTA format, the RefSeq accession number as well as the curated annotation done by experts. References or web sites are also mentioned.

Two different formats have been designed in *ProtAnnDB*: a tab delimited text format that can be imported in the Microsoft Office Excel format (http://www.microsoft.com/france/office/2007/programs/excel/overview.mspx) (Fig. 6) and a friendly user web interface format for each protein entry (Fig. 7). This presentation allows (i) checking the annotation of each protein separately and (ii) working with several proteins at the same time. In future versions of *ProtAnnDB*, it is planned to introduce other plant proteins such as those from rice and poplar since their genomes will been soon fully annotated. In addition, it is planned to allow querying the database with keywords related to sub-cellular localization and/or functional domains.

## Conclusion

Proteomic studies generate huge amounts of data consisting in lists of genes. Answering biological questions using these results is a great challenge. It is usually done collecting protein annotations from databases. However, protein annotation in databases can be incomplete or misleading since they are mainly inherited from results of sequence comparisons and do not usually take into account the presence of functional domains. Moreover, the available databases do not allow finding predictions of sub-cellular localization of proteins easily. *ProtAnnDB* has been designed as a new tool to facilitate the processing of proteomic data. Of course, it also allows processing of transcriptomic data which encounters the same type of problem. *ProtAnnDB* allows collecting results of prediction of sub-cellular localization and functional domains of *Arabidopsis* proteins by existing bioinformatics software. It should allow improving the biological interpretation of proteomic and transcriptomic data.

## Methods

*ProtAnnDB* contains all the AGI codes included in the TAIR8 database (http://www.arabidopsis.org/help/helppages/BLAST_help.jsp#datasets). Each AGI code is associated with the results of the predictions of sub-cellular localization and functional domains. *ProtAnnDB* has been filled using Perl scripts by a two-step procedure. As a first step, all the *Arabidopsis* protein sequences were submitted to the following programs: TargetP, SignalP, TMHMM, and Predotar for sub-cellular localization; PROSITE (ps-scan.pl) for functional domains. InterPro and PFAM domains were imported from the TAIR8_all.domain file available at TAIR (ftp://ftp.arabidopsis.org/home/tair/Proteins/Domains/). Predictions of GPI anchors have been imported from the Aramemnon database. As a second step, all the results were organized in a MySQL database, which can be queried with a friendly user web interface written in PHP.

## Supplementary Materials



## Figures and Tables

**Table 1.** **Evaluation of different methods for the recovery of plant secreted proteins.** Efficiency of the classical methods used to assess the purity of subcellular fractions (Methods for purity control) is compared to the results of the bioinformatic analysis of the subcellular localization of the proteins identified by mass spectrometry and bioinformatics. Estimated fraction purity refers to results of classical methods of analysis whereas cell wall protein fraction purity (ratio between number of predicted secreted proteins and total number of proteins) refers to results of bioinformatic analysis. Negative purity control for the cell wall fraction (–); positive purity control for the cell wall fraction (+); 1D-E: mono-dimensional gel electrophoresis; ADH: alcohol dehydrogenase; G-6-PDH: glucose-6-P dehydrogenase; MDH: malate dehydrogenase.

**Plant material**	**Preparation of the cell wall fraction**	**Method for purity control**	**Estimated fraction purity**	**Number of predicted secreted proteins**	**Total number of proteins**	**Number of proteins with uncertain sub-cellular localization**	**Cell wall protein fraction purity**	**Reference**
**Non-destructive methods**								
*A. thaliana* cell suspension cultures	washings with salt solutions	**Microscopy**	>60%	51	96	0	53.1%	Borderies et al.^23^
*A. thaliana* cell suspension cultures	culture medium	**Enzymology** (G-6-PDH: − ; ADH: −) **Immunology** (OEE, AnnAt1, cyclin D, GST: −; myrosinase: +)	>99%	9	13	1	69.2%	Oh et al.^24^
*A. thaliana* and *O. sativa* leaves	intercellular fluids	**Enzymology** (G-6-PDH: −)	>99%	6	13	3	46.1%	Haslam et al.^25^
*A. thaliana* leaves	intercellular fluids	**Enzymology** (MDH: −)	>90%	87	93	0	93.5%	Boudart et al.^26^
**Destructive methods**								
*A. thaliana* cell suspension cultures	water extraction; 10% glycerol sedimentation	**Enzymology** (callose synthase: −) **Immunology** (plasma membrane and chloroplasts: −; pectins: +)	>99%	24	75	2	32.0%	Chivasa et al.^27^
*A. thaliana* cell suspension cultures	salt solution containing 10% glycerol; extensive washings; CaCl_2_ final washing	**Microscopy**	>90%	89	792	0	12.6%	Bayer et al.^28^
*M. sativa* stems	filtration and extensive washing	different protein patterns after 1D-E analysis of different fractions during the purification procedure	qualitative	25	74	9	33.8%	Watson et al.^29^
*A. thaliana* etiolated hypocotyls	low salt buffer; increasing sucrose density sedimentation; extensive washing	none	not determined	73	99	4	73.7%	Feiz et al.^1^

**Table 2.** Number of *Arabidopsis* proteins annotated as LRXs in various databases and by Baumberger et al.^13^

	**LRX**	**Extensin-like**	**Hypothetical protein**	**Unknown protein**	**Disease resistance protein**	**Not found**
NCBI	14					
TAIR	14					
TIGR	14					
MIPS	1	6	3	1	2	1
Baumberger et al.^13^	11					

**Table 3.** Functional domains found using InterProScan and PROSITE in *Arabidopsis* proteins annotated as LRXs in databases. **IPR001611**: leucine-rich repeat; **PF00560**: LRR_1; IPR013210: leucine-rich repeat, N-terminal; **PF08263**: LRR_NT; **PS50099**: PRO_RICH proline-rich region profile; **IPR003882**: pistil-specific extensin-like protein; **PR01218**: PSTLEXTENSIN; IPR003883: extensin-like protein; **PF02095**: Extensin_1; PR01217: PRICHEXTENSN.

**AGI accession number**	**Annotation by Baumberger et al.^13^**	**Leucine-rich repeat domains**			**Proline-rich domains**		
		**IPR001611 PF00560**	**IPR013210 PF08263**	**PS50099**	**IPR003882 PR01218**	**IPR003883 PF02095**	**PR01217**
At1g12040	AtLRX1	1	1	1			1
At1g49490	AtPEX2	1	1	1	1		
At1g62440	AtLRX2	1		1		1	
At2g15880	AtPEX3	1		1	1		1
At2g19780		1	1				
At3g19020	AtPEX1	1	1	1	1		1
At3g22800	AtLRX6	1	1	1	1		1
At3g24480	AtLRX4	1	1	1			1
At4g06744		1					
At4g13340	AtLRX3	1	1	1	1		1
At4g18670	AtLRX5	1	1	1			1
At4g29240		1	1				
At4g33970	AtPEX4	1	1	1			1
At5g25550	AtLRX7	1	1	1			

**Table 4.** Cell wall-related protein families of *Arabidopsis* annotated by experts.

**Cell wall protein families**	**Curated annotation**
**Prolyl-4-hydroxylases**	http://cellwall.genomics.purdue.edu/families/4-6-5.html
**Nucleotide-sugar interconversion pathway**	http://cellwall.genomics.purdue.edu/families/1-1.html Reiter and Vanzin^30^
**GT8**	http://cellwall.genomics.purdue.edu/families/2-3-1.htmlhttp://www.cazy.org/fam/GT8.html
**GT31**	http://cellwall.genomics.purdue.edu/families/2-3-5.htmlhttp://www.cazy.org/fam/GT31.html
**GT34**	http://cellwall.genomics.purdue.edu/families/2-3-4.htmlhttp://www.cazy.org/fam/GT34.html
**GT37**	http://cellwall.genomics.purdue.edu/families/1-1.htmlhttp://www.cazy.org/fam/GT37.html
**GT47**	http://cellwall.genomics.purdue.edu/families/2-3-2.htmlhttp://www.cazy.org/fam/GT47.html
**GT77**	http://www.cazy.org/fam/GT77.html Egelund et al.^31^
**GT2** (cellulose synthases)	http://cellwall.genomics.purdue.edu/families/2-2.htmlhttp://www.cazy.org/fam/GT2.html
**GT2** (cellulose synthases-like)	http://cellwall.genomics.purdue.edu/families/2-2.htmlhttp://www.cazy.org/fam/GT2.html
**GT48** (callose synthases)	http://cellwall.genomics.purdue.edu/families/2-4.htmlhttp://www.cazy.org/fam/GT48.html
**Vesicle trafficking** (emp24/gp25L/p24 family protein)	http://cellwall.genomics.purdue.edu/families/3-1.html
**GH9** (endoglucanases)	http://cellwall.genomics.purdue.edu/families/4-3-2-1.htmlhttp://www.cazy.org/fam/GH9.html
**GH16** (xyloglucan endotransglycosylases/hydrolases) (XTHs)	http://labs.plantbio.cornell.edu/xth/genes.htm
**GH17**	http://cellwall.genomics.purdue.edu/families/4-3-2-2.htmlhttp://www.cazy.org/fam/GH17.html
**GH18** (yieldins)	http://cellwall.genomics.purdue.edu/families/4-1-2.htmlhttp://www.cazy.org/fam/GH18.html
**GH28** (polygalacturonases)	http://cellwall.genomics.purdue.edu/families/4-3-3.htmlhttp://www.cazy.org/fam/GH28.html
**GH35** (beta-galactosidases)	http://cellwall.genomics.purdue.edu/families/4-3-1-1.htmlhttp://www.cazy.org/fam/GH35.html
**CE8** (pectin methylesterases)	http://cellwall.genomics.purdue.edu/families/4-5-1.htmlhttp://www.cazy.org/fam/CE8.html
**CE13** (pectin acylesterases)	http://cellwall.genomics.purdue.edu/families/4-5-2.htmlhttp://www.cazy.org/fam/CE13.html
**PL1** (pectate lyases)	http://cellwall.genomics.purdue.edu/families/4-4-1.htmlhttp://www.cazy.org/fam/PL1.html
**PL4** (rhamnogalacturonan lyases)	http://cellwall.genomics.purdue.edu/families/4-4-2.htmlhttp://www.cazy.org/fam/PL4.html
**Expansins**	http://www.bio.psu.edu/expansins/index.htm
**arabinogalactan proteins** (AGPs)	Schultz et al.^32^ Johnson et al.^14^ Van Hengel and Roberts^33^ Liu and Mehdy^34^
**fasciclin AGPs** (FLAs)	Schultz et al.^32^
**GPI-anchored peptide** (GAPEP) **family**	http://cellwall.genomics.purdue.edu/families/6-4-10.html
**COBRA-like proteins**	Roudier et al.^35^
**leucine-rich repeat extensins** (LRXs)	Baumberger et al.^13^
**Hyp/Pro-rich proteins (H/PRP)**	Fowler et al.^36^
**Extensins**	Johnson et al.^14^
**lignin toolbox**	Raes et al.^37^
**peroxidases**	http://peroxibase.isb-sib.ch/index.php
**laccases**	Pourcel et al.^38^ McCaig et al.^39^
**SKU-like proteins** (multi-copper oxidases)	Jacobs and Roe^40^
**phytocyanins**	Nersissian and Shipp^15^
**subtilases**	http://csbdb.mpimp-golm.mpg.de/csbdb/dbcawp/psdb/pub/sgenes.html
**peptidases**	http://merops.sanger.ac.uk/
**peptidase inhibitors**	http://merops.sanger.ac.uk/
**receptor-like kinases**	Shiu and Bleecker^41^
