# Supplementary material for: Bioinformatics as a Tool for Assessing the Quality of Sub-Cellular Proteomic Strategies and Inferring Functions of Proteins: Plant Cell Wall Proteomics as a Test Case
Source: Bioinform Biol Insights. 2009 Feb 18;3:15–28. doi: 10.4137/bbi.s2065 (PMC2808182; doi:10.4137/bbi.s2065)
Supplement: Supplementary file 1 [file Supp1.pdf]

Table 1, supplementary data. Annotation of Arabidopsis LRXs and related proteins in databases.

Results from query with "extensin AND "repeat" as key words at NCBI Map Viewer (http://www.ncbi.nlm.nih.gov/mapview/map_search.cgi?chr=arabid.inf). Twelve genes labelled with pink boxes were found with with search. The 11 LRX genes annotated by Baumberger et al. (2003) are labelled with violet boxes. Functional domains were found using InterProScan (http://www.ebi.ac.uk/Tools/InterProScan/) and ScanProsite (http://www.expasy.ch/tools/scanprosite/). Names of domains (IPR: InterProScan; PF: PFAM; PR: SPRINT; PS: PROSITE) are indicated as well as the corresponding scores and positions on the amino acid sequence between square brackets.

	Annotations in databases	Interproscan and Prosite results		Annotation by Baumberger et al. (2003)
At1g12040	**NCBI**	**IPR001611: Leucine-rich repeat**		AtLRX1
	**LRX1 (LEUCINE-RICH REPEAT/EXTENSIN 1)**<br>similar to LRX2 (LEUCINE-RICH REPEAT/EXTENSIN 2), protein binding / structural constituent of cell wall [Arabidopsis thaliana] (TAIR:AT1G62440.1)<br>contains InterPro domain Leucine-rich repeat, N-terminal (IPR013210)<br>contains InterPro domain Leucine-rich repeat (IPR001611)	PF00560: LRR_1	6000.000322506199 [148-170]T,<br>32.9999983317489 [172-191]T,<br>2100.0003726265404 [243-265]T,<br>3500.00043291567 [267-289]T,<br>39.9999971482335 [291-313]T	
		**IPR013210: Leucine-rich repeat, N-terminal**<br>PF08263: LRR_NT	0.04300000526153389 [51-90]T	
	**TAIR**	**unintegrated**<br>PR01217: PRICHEXTENSN	4.8E-14 [383-395]T, 4.8E-14 [406-427]T, 4.8E-14 [428-444]T, 4.8E-14 [457-482]T	
	**LEUCINE-RICH REPEAT/EXTENSIN 1, LRX1**<br>encodes a a chimeric leucine-rich repeat/extensin protein that regulates root hair morphogenesis and elongation. Null mutants develop root hairs that frequently abort, swell, or branch. Gene is expressed in root hair cells and protein is specifically localized in the wall of the hair proper.<br><br>Leucine-rich repeat (N-terminal:IPR013210)<br>Leucine-rich repeat;Molecular Function: protein bi (IR001611)	PS50099: PRO_RICH Proline-rich region profile	score = 104.855 [383 - 741]	
	**TIGR**<br>**leucine-rich repeat family protein / extensin family protein (LRX1)**<br>similar to extensin-like protein [Lycopersicon esculentum] gi|5917664|gb|AAD55979<br>contains leucine-rich repeats (PF00560)<br>contains proline rich extensin domains (IPR002965)			
	**MIPS**<br>**leucine-rich repeat/extensin 1 (LRX1)**			
At1g49490	**NCBI**	**IPR001611: Leucine-rich repeat**		AtPEX2
	**leucine-rich repeat family protein / extensin family protein**<br>similar to leucine-rich repeat family protein / extensin family protein [Arabidopsis thaliana] (TAIR:AT3G19020.1)<br>contains InterPro domain Leucine-rich repeat, N-terminal (IPR013210)<br>contains InterPro domain Pistil-specific extensin-like protein (IPR003882)<br>contains InterPro domain Leucine-rich repeat (IPR001611)	PF00560: LRR_1	1899.9996417184002 [180-202]T<br>3399.99972437719 [250-272]T<br>6000.000322506199 [274-296]T<br>58.999994304626 [298-320]T	
		**IPR003882: Pistil-specific extensin-like protein**<br>PR01218: PSTLEXTENSIN	1.4E-5 [530-553]T 1.4E-5 [565-583]T	
		**IPR013210: Leucine-rich repeat, N-terminal**<br>PF08263: LRR_NT	2.00000006595059 [59-98]T	
	**TAIR**<br>**leucine-rich repeat family protein / extensin family protein**<br>similar to leucine-rich repeat family protein / extensin family protein [Arabidopsis thaliana] (TAIR:AT3G19020.1)<br>contains InterPro domain Leucine-rich repeat, N-terminal (IPR013210)<br>contains InterPro domain Pistil-specific extensin-like protein (IPR003882)<br>contains InterPro domain Leucine-rich repeat (IPR001611)	PS50099: PRO_RICH Proline-rich region profile	score = 86.183 [394 - 842]	
	**TIGR**<br>**leucine-rich repeat family protein / extensin family protein**<br>contains similarity to disease resistance protein GI:3894383 from [Lycopersicon esculentum]<br>contains leucine-rich repeats (PF00560)<br>contains proline rich extensin domains (IPR002965)			
	**MIPS**<br>**hypothetical protein**			

**At1g62440**	**NCBI**	**IPR001611: Leucine-rich repeat**			**AtLRX2**
	**LRX2 (LEUCINE-RICH REPEAT/EXTENSIN 2)**<br>similar to LRX1 (LEUCINE-RICH REPEAT/EXTENSIN 1), protein binding / structural constituent of cell wall [Arabidopsis thaliana] (TAIR:AT1G12040.1)	PF00560: LRR_1	0.022 [214-233]T, 4.1 [285-307]T, 0.029 [309-331]T, 0.029 [333-355]T		
		**IPR003883: Extensin-like protein**			
	contains InterPro domain Extensin-like protein (IPR003883)<br>contains InterPro domain Leucine-rich repeat, N-terminal (IPR013210)<br>contains InterPro domain Leucine-rich repeat (IPR001611)	PF02095: Extensin_1	5.9 [636-645]T, 63 [664-673]T, 2.9 [679-688]T, 22 [693-702]T, 6.6 [722-731]T, 22 [737-746]T	Extensins are plant cell-wall proteins; they can account for up to 20% of the dry weight of the cell wall. They are highly-glycosylated, possibly reflecting their interactions with cell-wall carbohydrates. Amongst their functions is cell wall strengthening in response to mechanical stress (e.g., during attack by pests, plant-bending in the wind, etc.). This repeat occurs within extensin-like proteins.	
	**TAIR**	PS50099: PRO_RICH Proline-rich region profile	score = 108.140 [425 - 824]		
	**LEUCINE-RICH REPEAT/EXTENSIN 2, LRX2**<br>encodes a paralog of LRX1 (LEUCINE-RICH REPEAT/EXTENSIN 1) which acts synergistically with LRX1 in root hair cell morphogenesis.<br>Leucine-rich repeat, N-terminal (IPR013210)<br>Extensin-like protein;Molecular Function: structur (IPR003883)<br>Leucine-rich repeat;Molecular Function: protein bi (IPR001611)				
	**TIGR**				
	**leucine-rich repeat family protein / extensin family protein**<br>similar to extensin-like protein [Lycopersicon esculentum] gi|5917664|gb|AAD55979<br>contains leucine-rich repeats (PF00560)<br>contains proline rich extensin domains (IPR002965)				
	**MIPS**				
	**putative extensin-like protein (gnl|PID|e1310400)**				

**At2g15880**	**NCBI**	**IPR001611: Leucine-rich repeat**			**AtPEX3**
	**leucine-rich repeat family protein / extensin family protein**<br>similar to leucine-rich repeat family protein / extensin family protein [Arabidopsis thaliana] (TAIR:AT4G33970.1)	PF00560: LRR_1	51.9999946268774 [299-321]T		
		**IPR003882: Pistil-specific extensin-like protein**			
	contains InterPro domain Pistil-specific extensin-like protein (IPR003882)<br>contains InterPro domain Leucine-rich repeat (IPR001611)	PR01218: PSTLEXTENSIN	4.1E-10 [514-537]T, 4.1E-10 [549-567]T, 4.1E-10 [663-684]T		
		**unintegrated**			
	**TAIR**	PR01217: PRICHEXTENSN	2.7E-11 [390-402]T, 2.7E-11 [404-420]T, 2.7E-11 [424-436]T, 2.7E-11 [442-463]T, 2.7E-11 [465-481]T		
	**leucine-rich repeat family protein / extensin family protein**<br>leucine-rich repeat family protein / extensin family protein; similar to leucine-rich repeat family protein / extensin family protein [Arabidopsis thaliana] (TAIR:AT4G33970.1)<br>contains InterPro domain Pistil-specific extensin-like protein (IPR003882)<br>contains InterPro domain Leucine-rich repeat (IPR001611)	PS50099: PRO_RICH Proline-rich region profile	score = 109.004 [394 - 722]		
	**TIGR**				
	**leucine-rich repeat family protein / extensin family protein**<br>similar to extensin-like protein [Lycopersicon esculentum] gi|5917664|gb|AAD55979<br>contains leucine-rich repeats (PF00560)<br>contains proline rich extensin domains (IPR002965)				
	**MIPS**				
	**unknown protein**				

Gene	Annotation	Domain	Score	Name
**At2g19780**	**NCBI**	**IPR001611: Leucine-rich repeat**		**none**
	**leucine-rich repeat family protein / extensin family protein**	PF00560: LRR_1	4.599999811982681 [134-156]T,<br>20.0000020319536 [158-178]T,<br>5.90000024020674 [182-201]T,<br>6099.99977126217 [205-224]T,<br>45.000003473935095 [301-323]T	
	similar to leucine-rich repeat family protein / extensin family protein [Arabidopsis thaliana] (TAIR:AT4G29240.1)	**IPR013210: Leucine-rich repeat, N-terminal**		
	contains InterPro domain Leucine-rich repeat, N-terminal (IPR013210)	PF08263: LRR_NT	8.29999581180007E-5 [68-107]T	
	contains InterPro domain Leucine-rich repeat (IPR001611)			
	**TAIR**			
	**leucine-rich repeat family protein / extensin family protein**			
	similar to leucine-rich repeat family protein / extensin family protein [Arabidopsis thaliana] (TAIR:AT4G29240.1)			
	similar to hypothetical protein [Vitis vinifera] (GB:CAN62837.1)			
	contains InterPro domain Leucine-rich repeat, N-terminal (IPR013210)			
	contains InterPro domain Leucine-rich repeat (IPR001611)			
	**TIGR**			
	**leucine-rich repeat family protein / extensin family protein**			
	contains leucine rich-repeat (LRR) domains (PF00560, IPR001611)			
	similar to leucine-rich repeat/extensin 1 (GI:13809918) [Arabidopsis thaliana]			
	contains similarity to disease resistance protein [Lycopersicon esculentum] gi|3894383|gb|AAC78591			
	**MIPS**			
	**putative disease resistance protein**			

Gene	Annotation	Domain	Score	Name
**At3g19020**	**NCBI**	**IPR001611: Leucine-rich repeat**		**AtPEX1**
	**leucine-rich repeat family protein / extensin family protein**	PF00560: LRR_1	4000.00081278147 [193-215]T,<br>7300.001901602701 [263-285]T,<br>3800.00071256818 [287-309]T	
	similar to leucine-rich repeat family protein / extensin family protein [Arabidopsis thaliana] (TAIR:AT1G49490.1)	**IPR003882: Pistil-specific extensin-like protein**		
	contains InterPro domain Leucine-rich repeat, N-terminal (IPR013210)	PR01218: PSTLEXTENSIN	8.7E-6 [675-698]T, 8.7E-6 [710-728]T, 8.7E-6 [811-832]T	
	contains InterPro domain Pistil-specific extensin-like protein (IPR003882)	**IPR013210: Leucine-rich repeat, N-terminal**		
	contains InterPro domain Leucine-rich repeat (IPR001611)	PF08263: LRR_NT	5.3000002124871 [72-111]T	
	**TAIR**	**unintegrated**		
	**leucine-rich repeat family protein / extensin family protein**	PR01217: PRICHEXTENSN	6.4E-9 [406-418]T, 6.4E-9 [423-444]T, 6.4E-9 [446-462]T, 6.4E-9 [470-487]T	
	similar to leucine-rich repeat family protein / extensin family protein [Arabidopsis thaliana] (TAIR:AT1G49490.1)			
	contains InterPro domain Leucine-rich repeat, N-terminal (IPR013210)			
	contains InterPro domain Pistil-specific extensin-like protein (IPR003882)	PS50099: PRO_RICH Proline-rich region profile	score = 132.344 [407 - 954]	
	contains InterPro domain Leucine-rich repeat (IPR001611)			
	**TIGR**			
	**leucine-rich repeat family protein / extensin family protein**			
	similar to extensin-like protein [Lycopersicon esculentum] gi|5917664|gb|AAD55979			
	contains leucine-rich repeats (PF00560)			
	contains proline rich extensin domains (IPR002965)			
	**MIPS**			
	**hypothetical protein**			

Gene	Annotation	Domain	Score	Name
**At3g22800**	**NCBI**	**IPR001611: Leucine-rich repeat**		**AtLRX6**
	**leucine-rich repeat family protein / extensin family protein**	PF00560: LRR_1	2200.0002175608097 [148-170]T,<br>38.999996884611306 [172-194]T,<br>1499.99977583351 [268-290]T,<br>32.9999983317489 [292-314]T	
	similar to leucine-rich repeat family protein / extensin family protein [Arabidopsis thaliana] (TAIR:AT3G24480.1)	**IPR003882: Pistil-specific extensin-like protein**		
	similar to leucine-rich repeat family protein / extensin family protein [Arabidopsis thaliana] (TAIR:AT4G13340.1)	PR01218: PSTLEXTENSIN	1.2E-5 [400-423]T, 1.2E-5 [435-453]T	
	contains InterPro domain Leucine-rich repeat, N-terminal (IPR013210)	**IPR013210: Leucine-rich repeat, N-terminal**		
	contains InterPro domain Pistil-specific extensin-like protein (IPR003882)	PF08263: LRR_NT	0.0989999902584295 [51-90]T	
	contains InterPro domain Leucine-rich repeat (IPR001611)	**unintegrated**		
	**TAIR**	PR01217: PRICHEXTENSN	3.6E-14 [374-386]T, 3.6E-14 [386-407]T, 3.6E-14 [414-430]T	
	**leucine-rich repeat family protein / extensin family protein**			
	similar to leucine-rich repeat family protein / extensin family protein [Arabidopsis thaliana] (TAIR:AT4G13340.1)			
	contains InterPro domain Leucine-rich repeat, N-terminal (IPR013210)	PS50099: PRO_RICH Proline-rich region profile	score = 52.470 [376 - 467]	
	contains InterPro domain Pistil-specific extensin-like protein (IPR003882)			
	contains InterPro domain Leucine-rich repeat (IPR001611)			
	**TIGR**			
	**leucine-rich repeat family protein / extensin family protein**			
	similar to extensin-like protein [Lycsimilar to extensin-like protein [Lycopersicon esculentum] gi|5917664|gb|AAD55979			
	contains leucine-rich repeats (PF00560)			
	contains proline rich extensin domains (IPR002965)			
	**MIPS**			
	**hypothetical protein**			

**At3g24480**	**NCBI**	**IPR001611: Leucine-rich repeat**		**AtLRX4**
	**leucine-rich repeat family protein / extensin family protein**<br>similar to leucine-rich repeat family protein /extensin family protein [Arabidopsis thaliana] (TAIR:AT4G18670.1)<br>similar to leucine-rich repeat family protein / extensin family protein [Arabidopsis thaliana] (TAIR:AT4G13340.1)<br>contains InterPro domain Leucine-rich repeat, N-terminal (IPR013210)<br>contains InterPro domain Leucine-rich repeat (IPR001611)	PF00560: LRR_1	2100.0003726265404 [195-214]T, 5099.9988243997805 [289-311]T, 3099.99945899369 [313-335]T	
		**IPR013210: Leucine-rich repeat, N-terminal**		
		PF08263: LRR_NT	0.0739999967908919 [74-113]T	
		**unintegrated**		
		PR01217: PRICHEXTENSN	5.4E-16 [411-432]T, 5.4E-16 [434-450]T, 5.4E-16 [456-473]T	
	**TAIR**			
	**leucine-rich repeat family protein / extensin family protein**<br>similar to leucine-rich repeat family protein / extensin family protein [Arabidopsis thaliana] (TAIR:AT4G18670.1)<br>similar to leucine-rich repeat family protein / extensin family protein [Arabidopsis thaliana] (TAIR:AT4G13340.1)<br>contains InterPro domain Leucine-rich repeat, N-terminal (IPR013210)<br>contains InterPro domain Leucine-rich repeat (IPR001611)	PS50099: PRO_RICH Proline-rich region profile	score = 34.144 [403 - 492]	
	**TIGR**			
	**leucine-rich repeat family protein / extensin family protein**<br>similar to extensin-like protein [Lycopersicon esculentum] gi|5917664|gb|AAD55979<br>contains leucine-rich repeats (PF00560)<br>contains proline rich extensin domains (IPR002965)			
	**MIPS**			
	disease resistance protein, putative			

**At4g06744**	**NCBI**	**IPR001611: Leucine-rich repeat**		**none**
	**leucine-rich repeat family protein / extensin family protein**<br>Identical to Uncharacterized protein At4g06744 precursor [Arabidopsis Thaliana] (GB:Q8W3M4)<br>similar to leucine-rich repeat family protein [Arabidopsis thaliana] (TAIR:AT3G19320.1)<br>similar to leucine-rich repeat family protein [Arabidopsis thaliana] (TAIR:AT1G49750.1)<br>similar to Leucine-rich repeat, plant specific [Medicago truncatula] (GB:ABD32635.1)<br>contains InterPro domain Leucine-rich repeat (IPR001611)	PF00560: LRR_1	2700.00035356384 [143-165]T, 4100.000492084209 [167-189]T, 8199.9995664553 [215-238]T, 5200.00089003336 [264-286]T, 48.9999951782359 [314-335]T	
	**TAIR**			
	**leucine-rich repeat family protein / extensin family protein**<br>Identical to Uncharacterized protein At4g06744 precursor [Arabidopsis Thaliana] (GB:Q8W3M4)<br>similar to leucine-rich repeat family protein [Arabidopsis thaliana] (TAIR:AT3G19320.1)<br>similar to Leucine-rich repeat, plant specific [Medicago truncatula] (GB:ABD32635.1)<br>contains InterPro domain Leucine-rich repeat (IPR001611)			
	**TIGR**			
	**leucine-rich repeat family protein / extensin family protein**<br>similar to leucine-rich repeat/extensin 1 (GI:13809918) {Arabidopsis thaliana}<br>contains PF00560: Leucine Rich Repeat domains			
	**MIPS**			
	**not found**			

**At4g13340**	**NCBI**	**IPR001611: Leucine-rich repeat**		**AtLRX3**
	**leucine-rich repeat family protein / extensin family protein**	PF00560: LRR_1	2100.0003726265404 [187-206]T, 7199.99952919347 [281-303]T, 1599.99977185865 [305-327]T	
	similar to leucine-rich repeat family protein / extensin family protein [Arabidopsis thaliana] (TAIR:AT3G24480.1)<br>similar to leucine-rich repeat family protein / extensin family protein [Arabidopsis thaliana] (TAIR:AT4G18670.1)	**IPR003882: Pistil-specific extensin-like protein**<br>PR01218: PSTLEXTENSIN	6.2E-5 [416-439]T, 6.2E-5 [449-467]T	
	contains InterPro domain Leucine-rich repeat, N-terminal (IPR013210)<br>contains InterPro domain Pistil-specific extensin-like protein (IPR003882)	**IPR013210: Leucine-rich repeat, N-terminal**<br>PF08263: LRR_NT	0.00230000061930563 [66-105]T	
	contains InterPro domain Leucine-rich repeat (IPR001611)	**unintegrated**		
	**TAIR**	PR01217: PRICHEXTENSN	1.0E-14 [392-413]T, 1.0E-14 [425-441]T, 1.0E-14 [443-460]T	
	**leucine-rich repeat family protein / extensin family protein**<br>similar to leucine-rich repeat family protein / extensin family protein [Arabidopsis thaliana] (TAIR:AT3G24480.1)			
	similar to leucine-rich repeat family protein / extensin family protein [Arabidopsis thaliana] (TAIR:AT4G18670.1)	PS50099: PRO_RICH Proline-rich region profile	score = 153.609 [394 - 758]	
	contains InterPro domain Leucine-rich repeat, N-terminal (IPR013210)<br>contains InterPro domain Pistil-specific extensin-like protein (IPR003882)<br>contains InterPro domain Leucine-rich repeat (IPR001611)			
	**TIGR**			
	**leucine-rich repeat family protein / extensin family protein**<br>similar to extensin-like protein [Lycopersicon esculentum] gi|5917664|gb|AAD55979<br>contains leucine-rich repeats (PF00560)<br>contains proline rich extensin domains (IPR002965)			
	**MIPS**			
	**extensin-like protein**<br>strong similarity to known protein : extensin-like protein, Zea mays, PIR2:S49915			

**At4g18670**	**NCBI**	**IPR001611: Leucine-rich repeat**		**AtLRX5**
	**leucine-rich repeat family protein / extensin family protein**	PF00560: LRR_1	3200.00074719642 [199-218]T, 3099.99945899369 [293-315]T, 4600.00044330866 [317-339]T	
	similar to leucine-rich repeat family protein / extensin family protein [Arabidopsis thaliana] (TAIR:AT3G24480.1)<br>similar to leucine-rich repeat family protein / extensin family protein [Arabidopsis thaliana] (TAIR:AT4G13340.1)	**IPR002951: Atrophin**<br>PR01222: ATROPHIN	2.1E-5 [410-438]T, 2.1E-5 [759-780]T	
	contains InterPro domain Leucine-rich repeat, N-terminal (IPR013210)<br>contains InterPro domain Leucine-rich repeat (IPR001611)	**IPR013210: Leucine-rich repeat, N-terminal**<br>PF08263: LRR_NT	0.0739999967908919 [78-117]T	
	contains InterPro domain Atrophin (IPR002951)	**unintegrated**		
	**TAIR**	PR01217: PRICHEXTENSN	2.4E-11 [402-423]T, 2.4E-11 [426-442]T, 2.4E-11 [446-463]T, 2.4E-11 [465-490]T	
	**leucine-rich repeat family protein / extensin family protein**<br>similar to leucine-rich repeat family protein / extensin family protein [Arabidopsis thaliana] (TAIR:AT3G24480.1)			
	similar to leucine-rich repeat family protein / extensin family protein [Arabidopsis thaliana] (TAIR:AT4G13340.1)	PS50099: PRO_RICH Proline-rich region profile	score = 99.495 [406 - 837]	
	contains InterPro domain Leucine-rich repeat, N-terminal (IPR013210)	PS50324: SER_RICH Serine-rich region profile	score = 13.379 [418 - 596]	
	contains InterPro domain Leucine-rich repeat (IPR001611)	PS50316 HIS_RICH Histidine-rich region profile	score = 13.990 [628 - 674]	
	contains InterPro domain Atrophin (IPR002951)			
	**TIGR**			
	**leucine-rich repeat family protein / extensin family protein**<br>similar to extensin-like protein [Lycopersicon esculentum] gi|5917664|gb|AAD55979<br>contains leucine-rich repeats (PF00560)<br>contains proline rich extensin domains (IPR002965)			
	**MIPS**			
	**extensin-like protein**<br>strong similarity to known protein : extensin-like protein - maize, PIR2:S49915			

**At4g29240**	**NCBI**	**IPR001611: Leucine-rich repeat**		**none**
	**leucine-rich repeat family protein / extensin family protein**	PF00560: LRR_1	31.0000030991121 [139-161]T, 31.9999986882983 [163-183]T, 29.9999985646001 [187-206]T, 6899.99963379704 [210-232]T, 3099.99945899369 [306-328]T	
	similar to leucine-rich repeat family protein / extensin family protein [Arabidopsis thaliana] (TAIR:AT2G19780.1)	**IPR013210: Leucine-rich repeat, N-terminal**		
	contains InterPro domain Leucine-rich repeat, N-terminal (IPR013210)	PF08263: LRR_NT	0.016000002110419 [73-112]T	
	contains InterPro domain Leucine-rich repeat (IPR001611)			
	**TAIR**			
	**leucine-rich repeat family protein / extensin family protein**	PS50315 GLY_RICH Glycine-rich region profile	score = 14.253 [21 - 61]	
	similar to leucine-rich repeat family protein / extensin family protein [Arabidopsis thaliana] (TAIR:AT2G19780.1)			
	contains InterPro domain Leucine-rich repeat, N-terminal (IPR013210)			
	contains InterPro domain Leucine-rich repeat (IPR001611)			
	**TIGR**			
	**leucine-rich repeat family protein / extensin family protein**			
	contains PF00560: Leucine Rich Repeat domains			
	similar to leucine-rich repeat/extensin 1 (GI:13809918) [Arabidopsis thaliana]			
	**MIPS**			
	**extensin-like protein**			
**At4g33970**	**NCBI**	**IPR001611: Leucine-rich repeat**		**AtPEX4**
	**leucine-rich repeat family protein / extensin family protein**	PF00560: LRR_1	31.0000030991121 [325-347]T	
	similar to leucine-rich repeat family protein / extensin family protein [Arabidopsis thaliana] (TAIR:AT2G15880.1)	**IPR013210: Leucine-rich repeat, N-terminal**		
	contains InterPro domain Leucine-rich repeat, N-terminal (IPR013210)	PF08263: LRR_NT	0.52000001763605 [86-125]T	
	contains InterPro domain Leucine-rich repeat (IPR001611)	**unintegrated**		
	**TAIR**	PR01217: PRICHEXTENSN	4.0E-14 [418-430]T, 4.0E-14 [434-450]T, 4.0E-14 [454-466]T, 4.0E-14 [467-488]T, 4.0E-14 [490-506]T	
	**leucine-rich repeat family protein / extensin family protein**			
	similar to leucine-rich repeat family protein / extensin family protein [Arabidopsis thaliana] (TAIR:AT2G15880.1)			
	contains InterPro domain Leucine-rich repeat, N-terminal (IPR013210)	PS50099: PRO_RICH Proline-rich region profile	score = 82.898 [419 - 694]	
	contains InterPro domain Leucine-rich repeat (IPR001611)			
	**TIGR**			
	**leucine-rich repeat family protein / extensin family protein**			
	similar to extensin-like protein [Lycopersicon esculentum] gi|5917664|gb|AAD55979			
	contains leucine-rich repeats (PF00560)			
	contains proline rich extensin domains (IPR002965)			
	**MIPS**			
	**extensin-like protein**			
	strong similarity to known protein : extensin-like protein, Zea mays, PIR2:S49915			
**At5g25550**	**NCBI**	**IPR001611: Leucine-rich repeat**		**AtLRX7**
	**leucine-rich repeat family protein / extensin family protein**	PF00560: LRR_1	0.034 [172-191]T, 7.8 [218-240]T, 2.2 [267-289]T, 0.011 [291-313]T	
	similar to leucine-rich repeat family protein / extensin family protein [Arabidopsis thaliana] (TAIR:AT3G24480.1)	**IPR013210: Leucine-rich repeat, N-terminal**		
	contains InterPro domain Leucine-rich repeat, N-terminal (IPR013210)	PF08263: LRR_NT	6.5e-05 [51-90]T	
	contains InterPro domain Leucine-rich repeat (IPR001611)			
	**TAIR**			
	**leucine-rich repeat family protein / extensin family protein**	PS50099: PRO_RICH Proline-rich region profile	score = 16.337 [382 - 430]	
	similar to leucine-rich repeat family protein / extensin family protein [Arabidopsis thaliana] (TAIR:AT3G24480.1)			
	contains InterPro domain Leucine-rich repeat, N-terminal (IPR013210)			
	contains InterPro domain Leucine-rich repeat (IPR001611)			
	**TIGR**			
	**leucine-rich repeat family protein / extensin family protein**			
	similar to leucine-rich repeat/extensin 1 (GI:13809918) [Arabidopsis thaliana]			
	contains PF00560: Leucine Rich Repeat domains			
	**MIPS**			
	**extensin-like protein**			
	strong similarity to known protein : Pex1 extensin-like protein, Lycopersicon esculentum, EMBL:AF159296			

Table 2, supplementary data. Annotation of some cell wall proteins in databases.
Functional domains were found using InterProScan (http://www.ebi.ac.uk/Tools/InterProScan/) and ScanProsite (http://www.expasy.ch/tools/scanprosite/). Names of domains are as follows: IPR: InterProScan; PF: PFAM; PR: SPRINT; PTHR: PANTHER; PS: PROSITE; SSF: Superfamily. The corresponding scores are given as well as the positions on the amino acid sequence between square brackets.

	Annotations in databases	Interproscan and Prosite results			Annotation by experts
**Some extensin genes**					Johnson et al. (2003)
**At1g21310**	**NCBI**	**IPR006706: Extensin-like region**			extensin (ROOT SHOOT HYPOCOTYL DEFECTIVE) (RSH)
	**AtEXT3 (ROOT SHOOT HYPOCOTYL DEFECTIVE)**<br>contains InterPro domain Extensin-like region (IPR006706)	PF04554: Extensin-like region	1.9e-40 [9-220]T, 1.9e-33 [221-428]T	Extensins are homologous hydroxyproline-rich glycoproteins (HRGPs) found in the plant extracellular matrix. The key to the role of HRGPs in cell wall self-assembly and cell extension lies in their chemistry, which is dependent on extensive post-translatio	
	**TAIR**<br>**AtEXT3 (ROOT SHOOT HYPOCOTYL DEFECTIVE)**<br>contains InterPro domain Extensin-like region (IPR006706)	PS50099: PRO_RICH Proline-rich region profile	score = 95.519 [34 - 428]		
	**TIGR**<br>**proline-rich extensin-like family protein**<br>contains extensin-like region (PF04554)				
	**MIPS**<br>**extensin 3 (atExt3)(AtExt5)**				
**At1g26240**	**NCBI**	**IPR006706: Extensin-like region**			extensin
	**proline-rich extensin-like family protein**<br>contains InterPro domain Extensin-like region (IPR006706)	PF04554: Extensin-like region	1.10000150671643E-32 [10-143]T, 2.10000267834025E-23 [144-243]T, 4.0999959904575E-28 [244-362]T, 9.70001793430386E-24 [364-478]T		
	**TAIR**<br>**proline-rich extensin-like family protein**<br>contains InterPro domain Extensin-like region (IPR006706)	**unintegrated**<br>PR01217: PRICHEXTENSN	2.5E-10 [28-44]T, 2.5E-10 [50-62]T, 2.5E-10 [63-84]T, 2.5E-10 [88-104]T		
	**TIGR**	PS50099: PRO_RICH Proline-rich region profile	score = 150.151 [32 - 476]		
	**proline-rich extensin-like family protein**<br>similar to hydroxyproline-rich glycoprotein precursor gi|727264|gb|AAA87902<br>contains proline-rich extensin domains (IPR002965)	PS50328: TYR_RICH Tyrosine-rich region profile	score = 32.719 [28 - 470]		
	**MIPS**<br>**hypothetical protein**				
**At1g26250**	**NCBI**	**IPR006706: Extensin-like region**			extensin
	**proline-rich extensin, putative**<br>contains InterPro domain Extensin-like region (IPR006706)	PF04554: Extensin-like region	5.89998809405682E-29 [9-163]T, 1.99999636034536E-24 [164-273]T, 7.300005909151711E-16 [274-443]T		
	**TAIR**<br>**proline-rich extensin, putative**<br>contains InterPro domain Extensin-like region (IPR006706)	**unintegrated**<br>PR01217: PRICHEXTENSN	5.5E-10 [24-40]T, 5.5E-10 [44-56]T, 5.5E-10 [63-84]T, 5.5E-10 [88-104]T		
	**TIGR**	PS50099: PRO_RICH Proline-rich region profile	score = 131.825 [29 - 441]		
	**proline-rich extensin, putative**<br>similar to extensin gi|1165322|gb|AAB53156<br>contains proline-rich extensin domains (IPR002965)	PS50328: TYR_RICH Tyrosine-rich region profile	score = 37.973 [17 - 435]		
	**MIPS**<br>**hypothetical protein**				

**Some pollen Ole e1 allergen and extensin family proteins**					
**At1g78040**	**NCBI**	**IPR006041: Pollen Ole e 1 allergen and extensin**			**none**
	**pollen Ole e 1 allergen and extensin family protein**	PF01190: Pollen_Ole_e_I	9.70001793430386E-27 [32-153]T	These proteins are most probably secreted and consist of about 145 residues. There are six cysteines which are conserved in the sequence of these proteins. They seem to be involved in disulphide bonds	
	similar to unknown [Populus trichocarpa] (GB:ABK94215.1)				
	contains InterPro domain Pollen Ole e 1 allergen and extensin (IPR006041)				
	**TAIR**				
	**pollen Ole e 1 allergen and extensin family protein**				
	similar to unknown [Populus trichocarpa] (GB:ABK92993.1)				
	contains InterPro domain Pollen Ole e 1 allergen and extensin (IPR006041)				
	**TIGR**				
	**pollen Ole e 1 allergen and extensin family protein**				
	PF01190 : Pollen proteins Ole e I family				
	**MIPS**	**TonB box, conserved site**			
	**phosphoglycerate mutase 1 like protein**	PS00430: TONB_DEPENDENT_REC_1	0.0 [1-35]?		
	Prosite motif: 1-6 TonB-dependent receptor proteins signatures				
**At3g33790**	**NCBI**	**IPR006041: Pollen Ole e 1 allergen and extensin**			**AtAGP30**
	**pollen Ole e 1 allergen and extensin family protein**	PF01190: Pollen_Ole_e_I	9.09992432541697E-68 [112-239]T		
	similar to AGP31 (ARABINOGALACTAN-PROTEIN 31) structural constituent of cell wall (AT1G28290.1)				
	contains InterPro domain Pollen Ole e 1 allergen and extensin (IPR006041)				
	**TAIR**	PS50099: PRO_RICH Proline-rich region profile	score = 16.164 [30 - 103]		
	**pollen Ole e 1 allergen protein containing 14.6% proline residues**				
	similar to arabinogalactan protein (Daucus carota) (11322245)				
	contains PF01190: Pollen proteins Ole e I family				
	**TIGR**				
	**pollen Ole e 1 allergen and extensin family protein**				
	similar to arabinogalactan protein (Daucus carota) (11322245)				
	contains PF01190: Pollen proteins Ole e I family				
	**MIPS**				
	**putative proline-rich protein**				
**others**					
**At2g15770**	**NCBI**	**IPR003245: Plastocyanin-like**			**AtEN23**
	**glycine-rich protein**	PF02298: Cu_bind_like	0.026000001808627 [154-184]T		Nersissian and Shipp (2002)
	similar to glycine-rich protein (AT2G15780.1)	**IPR008972: Cupredoxin**			
	contains InterPro domain Cupredoxin (IPR008972)	SSF4953: Cupredoxins superfamily	4.0E-17 [141-258]T		
	contains InterPro domain Plastocyanin-like (IPR003245)				
	**TAIR**				
	**glycine-rich protein**	PS50324: SER_RICH Serine-rich region profile	score = 18.782 [21 - 136]		
	contains InterPro domain Cupredoxin (IPR008972)	PS50315: GLY_RICH Glycine-rich region profile	score = 8.908 [35 - 96]		
	contains InterPro domain Plastocyanin-like (IPR003245)				
	**TIGR**				
	**glycine-rich protein**				
	contains a domain related to blue copper-binding protein				
	similar to Stellacyanin (SP:P00302) {Rhus vernicifera}				
	**MIPS**				
	hypothetical protein				
**At1g15825**	**NCBI**	PS50099: PRO_RICH Proline-rich region profile	score = 24.463 [2 - 123]		**none**
	**hydroxyproline-rich glycoprotein family protein**				
	similar to leucine-rich repeat family protein / extensin family protein (AT3G19020.1)				
	**TAIR**				
	**hydroxyproline-rich glycoprotein family protein**				
	similar to leucine-rich repeat family protein / extensin family protein (AT3G19020.1)				
	**TIGR**				
	**hydroxyproline-rich glycoprotein family protein**				
	contains proline-rich extensin domains (IPR002965)				
	**MIPS**				
	**putative protein**				

**At1g12090**	**NCBI**<br>**ELP, EXTENSIN-LIKE PROTEIN**<br>similar to protease inhibitor/seed storage/lipid transfer protein (LTP) family protein (AT1G62510.1)<br>similar to extensin-like protein [Brassica napus] (AAK30571.1)<br>contains InterPro domain Plant lipid transfer protein/seed storage/trypsin-alpha amylase inhibitor (IPR003612)<br>contains InterPro domain Bifunctional inhibitor/plant lipid transfer protein/seed storage (IPR016140)<br>contains InterPro domain Plant lipid transfer protein and hydrophobic protein helical (IPR013770)<br>**TAIR**<br>**ELP, EXTENSIN-LIKE PROTEIN**<br>Plant lipid transfer protein/seed storage/trypsin (IPR003612<br>Bifunctional inhibitor/plant lipid transfer protein (IPR016140)<br>Plant lipid transfer protein and hydrophobic protein (IPR013770)<br>**TIGR**<br>**protease inhibitor/seed storage/lipid transfer protein (LTP) family protein**<br>similar to 14 kDa polypeptide [Catharanthus roseus] (407410)<br>contains Pfam protease inhibitor/seed storage/LTP family domain (PF00234)<br>**MIPS**<br>**pEARLI 1-like protein**<br>4Fe-4S ferredoxins, iron-sulfur binding region signature	**IPR003612: Plant lipid transfer protein/seed storage/trypsin-alpha amylase inhibitor**<br>PF00234: Tryp_alpha_amyl 6.09999977126231E-23 [55-137]T<br>**IPR001450: 4Fe-4S ferredoxin, iron-sulphur binding**<br>PF00198: 4FE4S_FERREDOXIN 0.0 [23-34]?	**none**

**AAK30571**<br>*Brassica napus*	**NCBI**<br>**extensin-like protein**<br>**98% identity with At1g12090**	**IPR003612: Plant lipid transfer protein/seed storage/trypsin-alpha amylase inhibitor**<br>PF00234: Tryp_alpha_amyl 1.3999989204989E-19 [55-137]T<br>**IPR001450: 4Fe-4S ferredoxin, iron-sulphur binding**<br>PF00198: 4FE4S_FERREDOXIN 0.0 [23-34]?	
